# Supplementary material for: Assessment tools for transition readiness in adolescents with inflammatory bowel disease: A scoping review
Source: PLoS One. 2025 Jan 7;20(1):e0317109. doi: 10.1371/journal.pone.0317109 (PMC11706401; doi:10.1371/journal.pone.0317109)
Supplement: S1 File — (PDF) [file pone.0317109.s004.pdf]

Search strategy

Chinese:

SU:(评估 or 评价 or 筛查 or 量表 or 问卷 or 清单 or 工具) and SU:(过渡期准备度 or 过渡准备) and SU:(青少年 or 患儿)

English

Step	Search strategy
#1	"Health Transition"[MeSH Terms] OR "Transition to Adult Care"[MeSH Terms] OR "Transitional Care"[MeSH Terms]
#2	"transition readiness"[Title/Abstract]
#3	#1 OR #2
#4	"adolescen*"[MeSH Terms]
#5	"children"[Title/Abstract] OR "young adult*"[Title/Abstract]
#6	#4 OR #5
#7	"access"[Title/Abstract] OR "measure"[Title/Abstract] OR "questionnaire"[Title/Abstract] OR "tool"[Title/Abstract] OR "scale"[Title/Abstract] OR "list"[Title/Abstract]
#8	#3 AND #6 AND #7
